# Oxymatrine inhibits the migration and invasion of hepatocellular carcinoma cells by reducing the activity of MMP-2/-9 via regulating p38 signaling pathway

**DOI:** 10.7150/jca.32875

**Published:** 2019-08-29

**Authors:** Kunlun Chen, Pengfei Zhu, Jianwen Ye, Yuan Liao, Zhicheng Du, Fangfang Chen, He Juanjuan, Shaojin Zhang, Wenlong Zhai

**Affiliations:** 1 Department of Hepatobiliary and Pancreatic Surgery, The First Affiliated Hospital of Zhengzhou University, Henan, 450052, P.R. China; 2 Department of Emergency, The First Affiliated Hospital of Zhengzhou University, Zhengzhou, Henan, 450052, P.R. China; 3 Department of Breast Surgery, The First Affiliated Hospital of Zhengzhou University, Zhengzhou, Henan, 450052, P.R. China; 4 Department of Ueology, The First Affiliated Hospital of Zhengzhou University, Zhengzhou, Henan, 450052, P.R. China

**Keywords:** oxymatrine, hepatocellular carcinoma, migration and invasion, matrix metalloproteinase, p38 signaling pathway

## Abstract

As one of the major alkaloid components in *Sophoraflavescensait* (kushen), oxymatrine has been used widely across the world in anti-inflammatory and anti-cancer therapies. However, the effect in the metastasis of hepatocellular carcinoma (HCC) and related mechanism(s) are still unclear. The present study aimed to investigate the anti-metastatic effect of oxymatrine on HCC cells. Oxymatrine could also inhibit the protein levels of MMP-2/-9 in a dose-dependent relationship. Moreover, oxymatrine reduces the activity of p38 signaling pathway via inhibiting the phosphorylation of p38. The inhibition effect of oxymatrine on the expression of MMP-2/-9 and the phosphorylated of p38 was also detected *in vivo*. Combined treatment with p38 signaling pathway inhibitor and oxymatrine may have a synergistic effect on MMP-2/-9 and invasion of HCC cells. Therefore, oxymatrine may have inhibited GBC invasiveness by reducing the expression of MMP-2/-9 via inhibiting the activity of p38 signaling pathway. As a potentially novel therapeutic drug, oxymatrine may play an important role in the treatment of HCC.

## Introduction

As one of the most common causes of cancer-related deaths worldwide, hepatocellular carcinoma (HCC) is responsible for lots of deaths per year 1. The prognosis of HCC is poor, because of its potent invasion and metastasis ability 2. Due to this, it is important to understand the underlying pathophysiological mechanisms of HCC, and develop therapeutic treatment for HCC.

Metastasis of cancer cells is a complex process, including degradation of extracellular matrix (ECM)^3^. Matrix metalloproteinases (MMPs) exert important roles in breakdown of the ECM. MMP-2/-9 are known to be essential for cancer cell invasion as they can degrade type IV, V, and VII collagens, and some other components of extracellular matrix and are intricately involved in cancer invasion and metastasis 1, 2, 4. Increasing p-p38 levels in HCC tissues were associated with tumor size and the formation of satellite tumors. High p-p38 expression could serve as a predictor for a poor survival for the patients with HCC 5.

Oxymatrine, one of the major alkaloid components in S*ophoraflavescensait* (kushen), has exhibited various pharmacological effects such as anti-hepatitis virus infection, anti-hepatic fibrosis, anti-inflammation, anti-anaphylaxis and other immune-regulation 6. Previous studies have also reported anti-cancer activity of oxymatrine 7.The present study analyzes the inhibition effect of oxymatrine on the migration and invasion of HCC cells, and found that oxymatrine could inhibit HCC cell migration and invasion by regulating MMP-2/-9 via inhibiting p38 signaling pathway.

## Materials and Methods

### Reagents

Oxymatrine (purity>98%) was purchased from Xi'an Undersun Biologics (Xi'an, China). The molecular formula of oxymatrine is C_15_H_24_N_2_O_2_ and its molecular weight is 264.36 6. The drug was dissolved in dimethyl sulfoxide (DMSO) to prepare a 20mmol/l stock solution and stored at -20℃ in the dark. Fetal bovine serum (FBS), penicillin, streptomycin and RPMI-1640 medium were ordered from Hyclone. Anti-MMP-2, MMP-9, p38 and p-p38 antibodies were ordered from Cell Signaling Technology (Beverly, Mass., USA). β-actin antibody was ordered from Santa Crus (Santa Cruz, Calif., USA).

### Cell culture

The human HCC cell line MHCC97H was obtained from the Liver Cancer Institute of Fudan University (Shanghai, China). Human HCC cell lines (HepG2, SMMC7721) were ordered from Shanghai Institute of Cell Biology (Shanghai, China). Cells were cultured in DMEM supplemented with 10% FBS, 100 U/ml penicillin, 100 µg/ml streptomycin, and 2 mmol/l glutamine. All cells were incubated at 37˚C with 5% CO_2_.

### Assays of cell viability

The colorimetric 3-(4,5-dimethylthiazol-2-yl) 2,5-diphenyltetrazolium bromide assay (MTT) was performed as previously described^6^. In brief, HCC cells (1×10^3^ cells/well) were seeded into 96-well plates and treated with various concentrations of oxymatrine (0, 0.1, 0.2, 0.4, 0.6, 0.8, and 1.0mg/ml) for 24h, 20μl of MTT solution (5mg/ml; Sigma-Aldrich, St. Louis, MO, USA) was added and then incubated for an additional 4 h at 37˚C. The formazan crystals were dissolved in 150μl DMSO (Sigma-Aldrich) and the absorbance of samples was measured at 490 nm using a micro-plate reader (Model 3550; Bio-Rad, Hercules, California, USA). The inhibitory rate of HCC cells proliferation was calculated according to the formula: (1-experimental absorbance value/control absorbance value) ×100%. Each experiment was conducted in triplicate.

### Cell migration and invasion assay

Invasion assay was measured using 24-well BioCoat Matrigel Invasion Chambers (Becton Dicknson, Bedford, MA) as described previously^8^. After treatment with oxymatrine for different time-intervals, cells were added into the inner well and cultured for 24 h. Cells that did not invade were removed, and the cells that invaded the matrigel were fixed with formalin, dyed with crystal violet, and invasiveness was measured. The migration assay was performed in a similar manner, except that the chambers were not coated with matrigel. For migration assay, chambers were not coated with matrigel, the other was did the same as invasion assay.

### Quantitative real-time RT-PCR

Total RNA was extracted with the RNeasy Mini kit (Invitrogen). cDNA was synthesized with SuperScript III Reverse Transcriptase (Invitrogen). Quantitative real-time RT-PCR (qRT-PCR) was did using SYBR Green II in accordance with the PrimeScript RT-PCR Kit protocol (TaKaRa) as described previously 9. The specific primers were used as described in our previous study 3. The relative gene copy number data of MMP-2/-9 was analyzed using the 2^-ΔΔCt^ method.

### Western blot analysis

After incubated with various concentrations of oxymatrine or SB203580 (Sigma-Aldrich, St Louis, MO; 10 μM), cells were suspended in 100μl of lysis buffer. After separating on 10 % SDS-polyacrylamide gel electrophoresis, proteins were transferred onto PVDF membranes. The membranes were subsequently blocked in defatted milk to reducing non-specific binding at 37˚C for 1 h and then incubated with different antibodies (MMP-2, MMP-9, p38, p-p38 and β-actin) in TBST containing 5% defatted milk at 4˚C overnight. Then membranes were incubated with corresponding second anti-body for 1 h at room temperature. The bands were measured with an enhanced chemiluminescence kit (Millipore, Billerica, MA, USA) and exposed by autoradiography. The densitometric analysis was carried out with Image J software (GE Healthcare, Buckinghamshire, UK).

### Experimental xenograft model

The animal protocol used in the present study was approved by the Institutional Animal Care and Use Committee of the First Affiliated Hospital of Zhengzhou University (Zhengzhou, China). Female immunodeficient BALB/c nude mice at 4 weeks of age (initial body weight, 16±2g) were purchased from Vital River Laboratory Animal Technology Co., Ltd. (Beijing, China). Mice were raised under pathogen‑free conditions at the Institute of Medicine, Zhengzhou University. Mice were maintained in a controlled environment (temperature, 25±2˚C; relative humidity, 70±5%; and a 12-h light/dark cycle) and fed standard laboratory food and water. GBC‑SD cells in the exponential phase of growth were resuspended in 200 µl of serum‑free culture medium at a density of 5x10^6^ cells. Subsequently, tumor xenografts were established by subcutaneous inoculation of these MHCC97H cells into the right flank of nude mice. The tumor bearing mice were treated with vehicle, oxymatrine (150 mg/kg) daily (i.v.) for 3 weeks. Tumor volumes were measured every 2 days. Tumor volume was calculated using calipers and the following formula: Tumor volume (mm^3^) = (length x width^2^)/2. At the end of the experiment, the tumor tissue was removed and measured.

### Statistical analysis

All data were expressed as the means ± SD. The statistical analysis was carried out using the SPSS 17.0 software (SPSS Inc., Chicago, Illinois, USA) to evaluate statistical differences. Student's t-test was used for comparisons between two groups and one-way or two-way analysis of variance was used to analyze statistical differences between groups under different conditions. *p*<0.05 was considered to be statistically significant. All statistical tests were two sided. We performed correlation analysis by Z test.

## Results

### Oxymatrine reduces the cellular viability of HepG2, MHCC97H and SMMC7721cells

**Figure 1:**
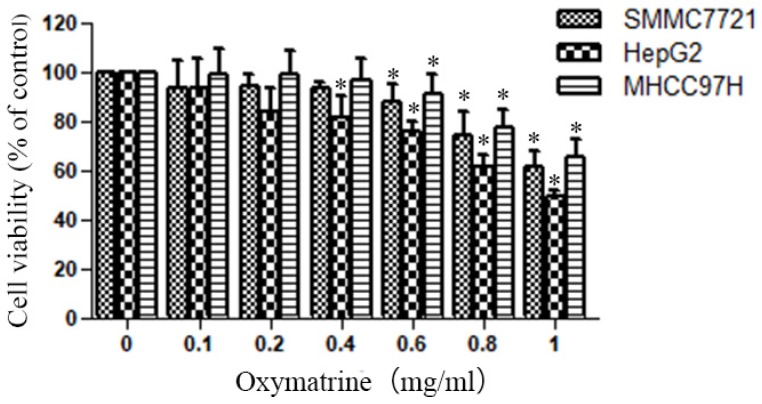
Oxymatrine reduces the cellular viability of HepG2, MHCC97H and SMMC7721 cells.

The cytotoxic effect of various concentrations of oxymatrine (0-1 mg/ml) on HepG2, MHCC97H and SMMC7721 cells was shown in Figure 1. The viability of HCC cells were inhibited by oxymatrine treatments in a dose-dependent manner, when the concentration was above 0.4 mg/ml. All the concentrations of oxymatrine in the invasion assay is below 0.4 mg/ml. We also found that oxymatrine did not affect the apoptosis of MHCC97H cells below 0.4 mg/ml.

Human HCC cell lines HepG2, MHCC97H and SMMC7721 were treated with oxymatrine for 24 h, and their viability was then measured using an MTT assay. Data are reported as means ± SD of three independent experiments. *p<0.05 vs control group. **p<0.01 vs control group.

### Oxymatrine reduces the metastasis ability of HCC cells

**Figure 2:**
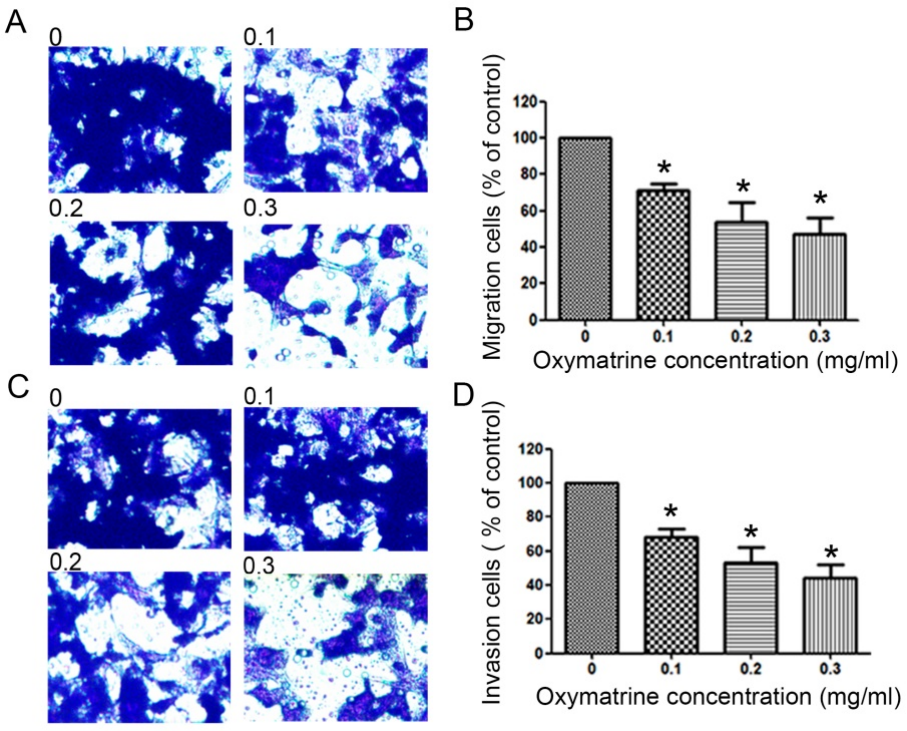
Anti-metastatic effects of oxymatrine in MHCC97H cells. (A) For the migration assay, MHCC97H cells were pre-incubated with various concentrations of oxymatrine (0, 0.1, 0.2 and 0.3 mg/ml) and analyzed. (B) The percent invasion rate was expressed as a percentage of the control (0 mg/ml). (C) For the invasion assay, MHCC97H cells were pre-incubated with various concentrations of oxymatrine (0, 0.1, 0.2 and 0.3 mg/ml) and analyzed. (D) The percent invasion rate was expressed as a percentage of the control (0 mg/ml). Cell spontaneous invasion in DMSO was designated as control. Values represent the means ± SD of three independent experiments. *p< 0.05 and **p< 0.01 vs control group.

To examine the invasive ability of HCC cells after oxymatrine treatment, transwell chambers coated with matrigel or not were used. The results suggested that the number of MHCC97H cells that migrated into the lower chamber was significantly reduced by oxymatrine under cytotoxic dose (Figure 2A and B). Oxymatrine significantly reduced the number of MHCC97H cells that passed through the matrigel member (Figure 2C and D). Figure 6 shows similar results in HepG2 and SMMC7721 cells.

### Oxymatrine suppresses the expression of MMP-2/-9 in HCC cells

**Figure 3:**
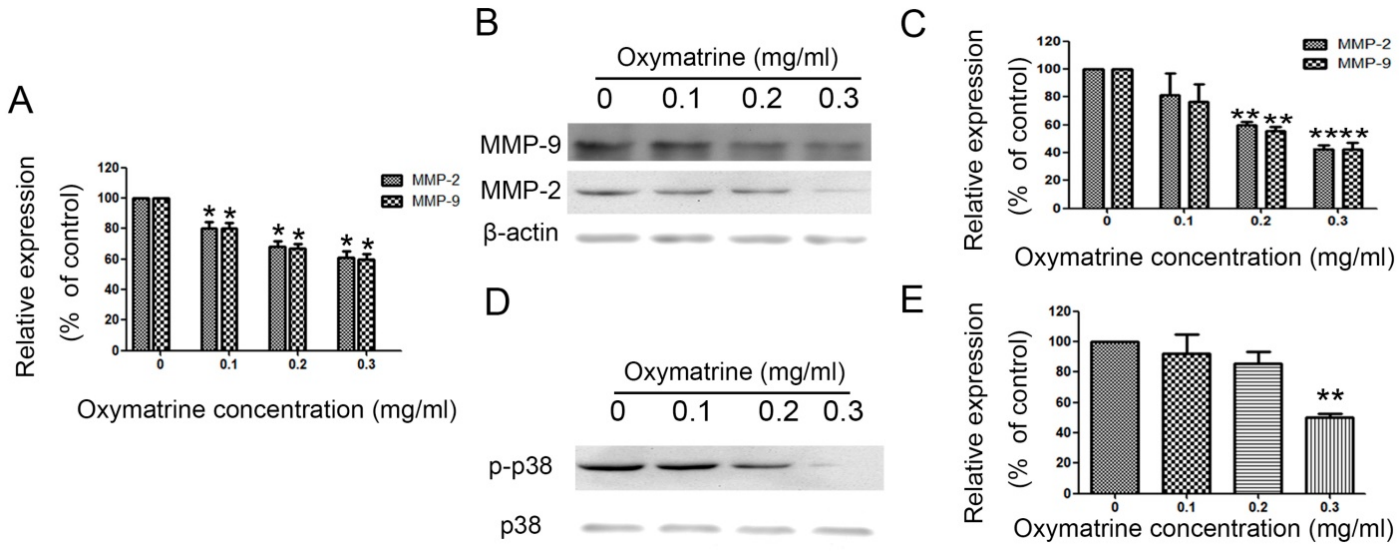
Oxymatrine suppresses the expression of MMP-2 /-9 and activity of p38 signaling pathway in MHCC97H cells.

MHCC97H cells were treated with oxymatrine (0, 0.1, 0.2 and 0.3 mg/ml) for 24 h, and then the RNA and protein expression was detected. Figure 3A shows the effect of oxymatrine on the RNA expression of MMP-2/-9. The inhibition effect of oxymatrine on the protein levels of MMP-2/-9 was positively correlated with the concentration (Figure 3B and 3C). Similar results were found in HepG2 and SMMC77211 cells (data was showed in Figure S1).

MHCC7H cells were treated with oxymatrine (0, 0.1, 0.2 and 0.3 mg/ml) for 24 h and then detected with real-time PCR and western blotting to analyze the RNA and protein levels of MMP-2 /-9. (A) Quantification of the RNA levels of MMP-2/-9 in MHCC97H cells. (B and C) Quantification of the protein levels of MMP-2/-9 in MHCC97H cells. (D) Protein levels of p38 and p- p38. (E) Phosphorylation densities of p38 were digitally scanned. Values represent the means ± SD of three independent experiments. *p< 0.05 and **p< 0.01 vs control group.

### Oxymatrine inhibit the metastasis of HCC cells via inhibiting p38 signaling pathway

We detected the effect of oxymatrine on the p38 signaling pathway in MHCC97H cells. We found that the activity of p38 signaling pathway was significantly reduced by oxymatrine (Figure 3D and 3E). Similar results were found in HepG2 and SMMC77211 cells (data was showed in Figure S1).

### Effect of p38 inhibitor (SB203580) and oxymatrine on the invasion and MMP-2/-9 expression of MHCC97H cells

**Figure 4:**
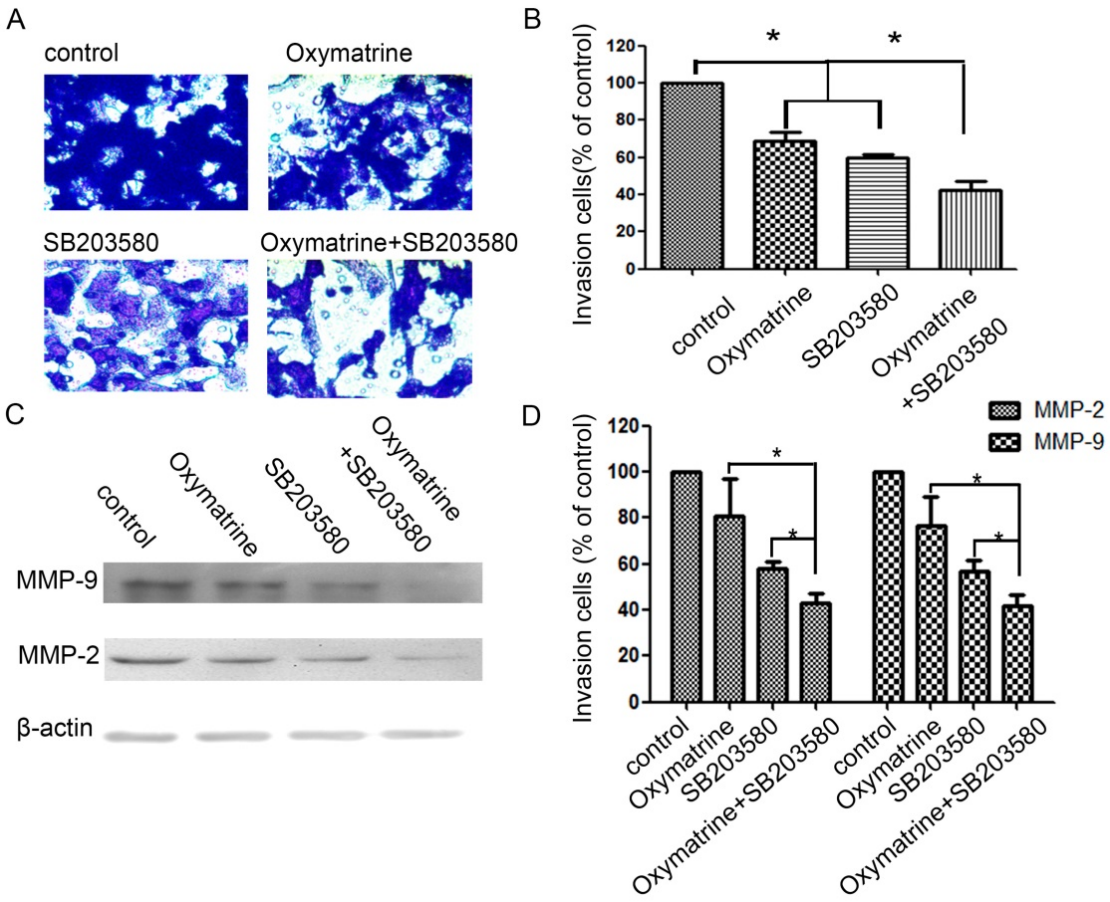
Effects of the p38 inhibitor SB203580 and oxymatrine on cell invasion and MMP-2/-9 expression in MHCC97H cells. (A) MHCC97H cells were pretreated with SB203580 and then incubated in the presence or absence of oxymatrine (0.1 mg/ml) for 24 h. Cellular invasiveness was measured using the transwell chamber invasion assay. (B) The percent invasion rate was expressed as a percentage of control. (C, D) The protein levels of MMP-2/-9 in MHCC97H cells treated with various concentrations of oxymatrine (0, 0.1, 0.2 and 0.3 mg/ml) and analyzed with western blotting. Values represent the means ± SD of three independent experiments. *p< 0.05 and **p< 0.01 vs control group.

In the study, cells were pretreated with SB203580, and then incubated in the presence or absence of oxymatrine (0.1 mg/ml) for 24 h (Figure 4). SB203580 and oxymatrine show a synergistic effect on the invasion and MMP-2/-9 expression of MHCC97H cells.

### Oxymatrine inhibits HCC cell growth and suppresses the expression levels of MMP-2 /-9, and the activity of p38 signaling pathway *in vivo*

**Figure 5:**
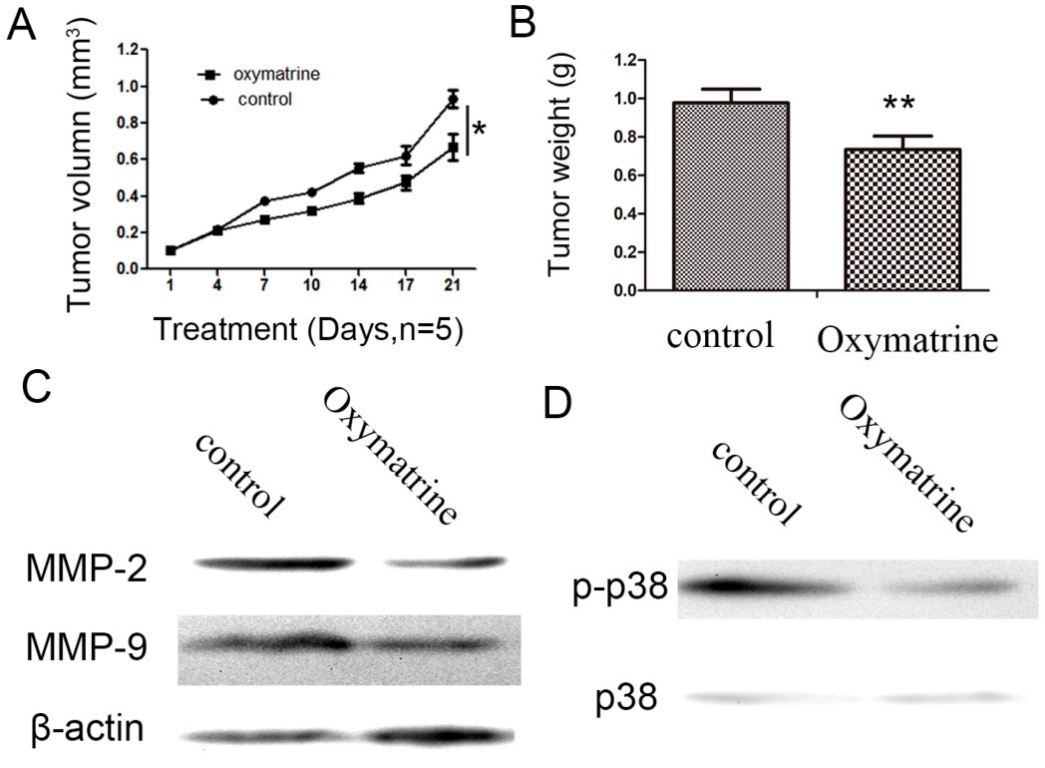
Oxymatrine inhibits the growth of tumor in MHCC97H xenografts. (A) Growth curves presented the volumes of tumor in BALB/c nude mice treated with PBS or oxymatrine. (B) The weight of tumor was measured in each group when the experiment was over. (C)The expression level of MMP-2 and MMP-9 in tumor tissues was detected by western blot. (D) The expression level of p38 and p-p38 in tumor tissues was detected by western blot. ^*^P<0.05 vs PBS (control). PBS, phosphate-buffered saline.

**Figure 6:**
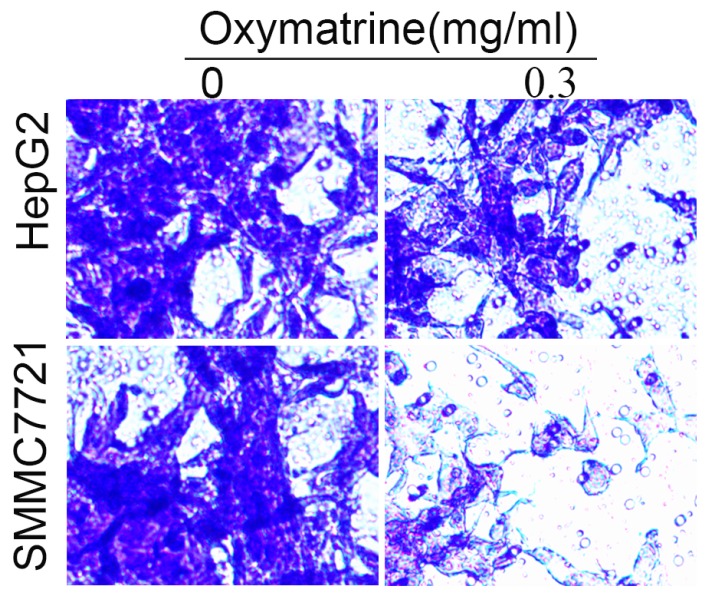
Oxymatrine inhibits the invasion of HepG2 and SMMC7721 cells.

As indicated in Figure 5, oxymatrine significantly inhibited the tumor weight and tumor growth rate (Figure 5A and B). Subsequently, the protein expression of MMP-2/-9 in the tumor xenograft tissues was detected by western blot analysis. As indicated in Figure 5C, treatment with oxymatrine suppressed the protein expression of MMP-2/-9 in tumor tissues, which was consistent with the results obtained *in vitro.* The inhibition effect of oxymatrine on the activity of p38 signaling pathway in tumor xenograft tissues (Figure 5D).

For the invasion assay, HepG2 and SMMC7721 cells were treated with oxymatrine (0.3 mg/ml) or not and analyzed.

HepG2 and SMMC77211 cells were treated with oxymatrine for 24 h or not, and then detected with western blotting to analyze the protein levels of MMP-2 /-9, p38 and p-p38. (A) The protein levels of MMP-2, MMP -9, p38 and p-p38 in HepG2 and SMMC77211 cells. (B) Quantification of the protein levels of MMP-2, MMP -9, p38 and p-p38 in HepG2 and SMMC77211 cells. Values represent the means ± SD of three independent experiments performed in triplicate. *p< 0.05 vs control group.

## Discussion

HCC is still a serious threat to public health problem worldwide 10, more than 50% of patients with HCC have metastases upon diagnosis. As a administration of the anti-tumor natural products, oxymatrine has been confirmed in various cancers 3, 6, 11-14. The anti-angiogenic effects of oxymatrine on human pancreatic cancer were confirmed in pancreatic cancer *in vitro* and *in vivo*, via regulating the activity of the NF-κB-mediated VEGF signaling pathway. But the effect of oxymatrine on HCC cells and related mechanism(s) is still unclear. The present study demonstrated the anti-metastatic ability of oxymatrine on HCC cells and related mechanisms.

To detect the anti-metastatic effect of oxymatrine, Transwell chamber assay had been taken. The present results revealed that oxymatrine could significantly inhibit the migration and invasion ability of HCC cells below toxic doses (less than 0.4 mg/ml). These results showed that the inhibition effect of oxymatrine on migration and invasion in MHCC97H cells was not due to cytotoxicity. The results are similar with previous studies among those oxymatrine could inhibit the invasion of various cancers, including glioblastoma, colorectal cancer, gallbladder cancer cells, gastric cancer and osteosarcoma 15-21.

Due to its aggressive nature, metastasis leads lots of cancer-related death among HCC patients. MMPs, especially MMP-2/-9, play important roles in breaking down the ECM 22, 23. Loss of the extracellular matrix (ECM) of blood or lymph vessels allows cancer cells to invade into the blood or lymphatic system and spread to other tissues and organs 3, 24. In our previous study, we found that MMP-2/-9 play important roles in the invasion of cancer cells 3. In gastric cancer, MMP-2/-9 are over-expressed and closely correlated with the metastasis of patients 24. In a previous study, oxymatrine has observed to inhibit the invasion of gallbladder cancer cells via inhibiting the expression of MMP-2/-9 18. To clarify the mechanism of action of oxymatrine on HCC invasion, the effect of oxymatrine on the expression of MMPs is detected. In the study, we found that oxymatrine could significantly inhibit the migration and invasion of HCC cells via reducing the expression of MMP-2/-9.

Accumulating evidence suggests that the interaction of signaling pathways is essential in cancer metastasis 25. Increasing p-p38 levels in HCC tissues were associated with tumor size and the formation of satellite tumors. High p-p38 expression could serve as a predictor for a poor survival for the patients with HCC 5. IFITM3 promotes HCC invasion and metastasis by regulating MMP-9 via inhibiting p38 signaling pathway 26. Naringin inhibits the invasion and migration of human glioblastoma cell via down-regulation of MMP-2/-9 expression and inactivation of p38 signaling pathway 26. In many studies, p38 signaling pathway plays an important role in the metastasis of HCC 25-27. To further explore the possible mechanism(s) of anti-metastatic effect of oxymatrine in HCC, we detected the activity of p38 signaling pathway in HCC cells. Oxymatrine was observed to inhibit the activity of p38 signaling pathway, which may have subsequently inhibited the expression of MMP-2/-9 in HCC cells. Combining oxymatrine and a p38 signaling pathway inhibitor (SB203580) caused significantly inhibition of HCC cell invasion via reducing MMP-2/-9, which further revealed the role of p38 signaling pathway in the anti-metastatic effect of oxymatrine.

In conclusion, this study showed the inhibition effect of oxymatrine on the migration and invasion of HCC cells. Furthermore, the inhibition effect of oxymatrine on MMP-2/-9 is attributed to blockade of the activity of p38 signaling pathway. These results uncover that oxymatrine may be a novel therapy in the metastasis of HCC.

## Supplementary Material

Supplementary figure.

## References

[1] Song J, Zhang X, Ge Q (2018). CRISPR/Cas9-mediated knockout of HBsAg inhibits proliferation and tumorigenicity of HBV-positive hepatocellular carcinoma cells. J Cell Biochem.

[2] Chen J, Xu X, Ling Q (2007). Role of Pittsburgh modified TNM criteria in prognosis prediction of liver transplantation for hepatocellular carcinoma. Chinese medical journal.

[3] Chen K, Zhang S, Ji Y, Li J (2013). Baicalein inhibits the invasion and metastatic capabilities of hepatocellular carcinoma cells via down-regulation of the ERK pathway. PLoS One.

[4] Gialeli C, Theocharis AD, Karamanos NK (2011). Roles of matrix metalloproteinases in cancer progression and their pharmacological targeting. FEBS J.

[5] Wang SN, Lee KT, Tsai CJ (2012). Phosphorylated p38 and JNK MAPK proteins in hepatocellular carcinoma. European journal of clinical investigation.

[6] Li M, Su BS, Chang LH (2014). Oxymatrine induces apoptosis in human cervical cancer cells through guanine nucleotide depletion. Anti-cancer drugs.

[7] Zhang Y, Piao B, Hua B (2011). Oxymatrine diminishes the side population and inhibits the expression of beta-catenin in MCF-7 breast cancer cells. Med Oncol.

[8] Wang L, Ma R, Kang Z (2014). Effect of IL-17A on the migration and invasion of NPC cells and related mechanisms. PLoS One.

[9] Yan X, Rui X, Zhang K (2015). Baicalein inhibits the invasion of gastric cancer cells by suppressing the activity of the p38 signaling pathway. Oncol Rep.

[10] Hiwatashi K, Ueno S, Sakoda M (2015). Problems of Long Survival Following Surgery in Patients with NonBNonC-HCC: Comparison with HBV and HCV Related-HCC. Journal of Cancer.

[11] Wu XS, Yang T, Gu J (2014). Effects of oxymatrine on the apoptosis and proliferation of gallbladder cancer cells. Anti-cancer drugs.

[12] Chen H, Zhang J, Luo J (2013). Antiangiogenic effects of oxymatrine on pancreatic cancer by inhibition of the NF-kappaB-mediated VEGF signaling pathway. Oncol Rep.

[13] He M, Jiang L, Li B (2017). Oxymatrine suppresses the growth and invasion of MG63 cells by up-regulating PTEN and promoting its nuclear translocation. Oncotarget.

[14] Dai Z, Wang L, Wang X (2018). Oxymatrine induces cell cycle arrest and apoptosis and suppresses the invasion of human glioblastoma cells through the EGFR/PI3K/Akt/mTOR signaling pathway and STAT3. Oncol Rep.

[15] Qian L, Li X, Ye P, Wang G (2018). Oxymatrine induces apoptosis and inhibits invasion in Gallbladder carcinoma via PTEN/PI3K/AKT pathway. Cytotechnology.

[16] Huang Y, Zhang J, Wang G (2018). Oxymatrine exhibits anti-tumor activity in gastric cancer through inhibition of IL-21R-mediated JAK2/STAT3 pathway. International journal of immunopathology and pharmacology.

[17] Ni Z, Yi J (2017). Oxymatrine induces nasopharyngeal cancer cell death through inhibition of PI3K/AKT and NFkappaB pathways. Molecular medicine reports.

[18] Wang X, Liu C, Wang J (2017). Oxymatrine inhibits the migration of human colorectal carcinoma RKO cells via inhibition of PAI-1 and the TGF-beta1/Smad signaling pathway. Oncol Rep.

[19] Guo B, Zhang T, Su J (2015). Oxymatrine targets EGFR(p-Tyr845) and inhibits EGFR-related signaling pathways to suppress the proliferation and invasion of gastric cancer cells. Cancer chemotherapy and pharmacology.

[20] Park SY, Kim JH, Lee YJ (2013). Surfactin suppresses TPA-induced breast cancer cell invasion through the inhibition of MMP-9 expression. Int J Oncol.

[21] Deng W, Sui H, Wang Q (2013). A Chinese herbal formula, Yi-Qi-Fu-Sheng, inhibits migration/invasion of colorectal cancer by down-regulating MMP-2/9 via inhibiting the activation of ERK/MAPK signaling pathways. BMC complementary and alternative medicine.

[22] Wu CY, Wu MS, Chen YJ (2007). Clinicopathological significance of MMP-2 and TIMP-2 genotypes in gastric cancer. Eur J Cancer.

[23] Liao Z, Zhang H, Fan P (2019). High PLK4 expression promotes tumor progression and induces epithelialmesenchymal transition by regulating the Wnt/betacatenin signaling pathway in colorectal cancer. Int J Oncol.

[24] Min J, Feng Q, Liao W (2018). IFITM3 promotes hepatocellular carcinoma invasion and metastasis by regulating MMP9 through p38/MAPK signaling. FEBS open bio.

[25] Chun SY, Kim S, Nam KS (2018). Anti-metastatic potential of a proton beam is regulated by p38 MAPK/c-Fos signaling pathway in TPA-treated HepG2 human hepatocellular carcinoma. Biomedicine & pharmacotherapy = Biomedecine & pharmacotherapie.

[26] Bai ZT, Wu ZR, Xi LL (2017). Inhibition of invasion by N-trans-feruloyloctopamine via AKT, p38MAPK and EMT related signals in hepatocellular carcinoma cells. Bioorganic & medicinal chemistry letters.

[27] Lin W, Zhong M, Liang S (2016). Emodin inhibits migration and invasion of MHCC-97H human hepatocellular carcinoma cells. Exp Ther Med.

