# Supplementary material for: Oxymatrine inhibits the migration and invasion of hepatocellular carcinoma cells by reducing the activity of MMP-2/-9 via regulating p38 signaling pathway
Source: J Cancer. 2019 Aug 29;10(22):5397–403. doi: 10.7150/jca.32875 (PMC6775708; doi:10.7150/jca.32875)
Supplement: Supplementary file 1 — Supplementary figure. [file jcav10p5397s1.pdf]

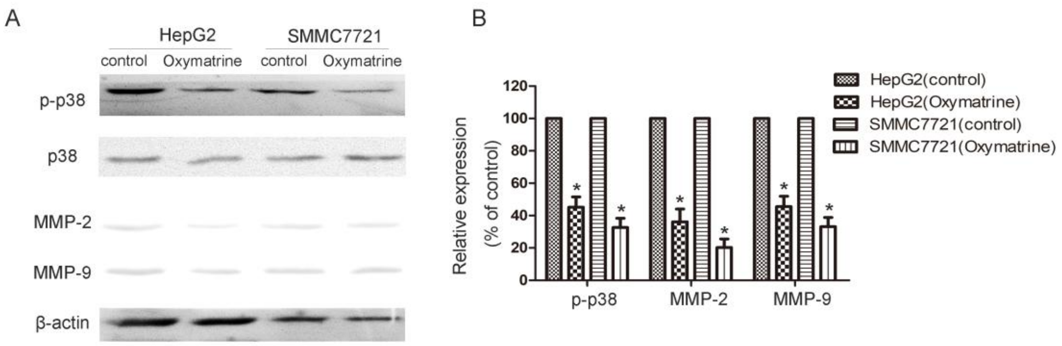

Figure S1. Oxymatrine suppresses the expression of MMP-2 /-9 and activity of p38 signaling pathway in HepG2 and SMMC77211 cells.

HepG2 and SMMC77211 cells were treated with oxymatrine for 24 h or not, and then detected with western blotting to analyze the protein levels of MMP-2 /-9, p38 and p-p38. (A)The protein levels of MMP-2, MMP -9, p38 and p-p38 in HepG2 and SMMC77211 cells. (B) Quantification of the protein levels of MMP-2, MMP -9, p38 and p-p38 in HepG2 and SMMC77211 cells. Values represent the means ± SD of three independent experiments performed in triplicate. *$p < 0.05$ vs control group.
